# The effect of repeated methotrexate injections on the quality of life of children with rheumatic diseases

**DOI:** 10.1007/s00431-018-3286-8

**Published:** 2018-11-17

**Authors:** Justin Jacobse, Wouter ten Voorde, Robert Rissmann, Jacobus Burggraaf, Rebecca ten Cate, Lenneke Schrier

**Affiliations:** 1 Department of Pediatric Rheumatology Willem-Alexander Children’s Hospital, Leiden University Medical Center, Albinusdreef 2, 2333 ZA Leiden, The Netherlands; 2 Centre for Human Drug Research, Zernikedreef 8, 2333 CL Leiden, The Netherlands; 3 Leiden Academic Center for Drug Research, Einsteinweg 55, 2333 CC Leiden, The Netherlands

**Keywords:** Fear of needles, Parental drugs, Biologicals, Juvenile idiopathic arthritis, Injection pain

## Abstract

In clinical practice, the burden of repeated injections in children with rheumatic disease receiving disease-modifying anti-rheumatic drugs is significant. To investigate the nature and extent of impact on the quality of life after repeated injections, we conducted a literature review. Two relevant papers were identified, both about children with juvenile idiopathic arthritis (JIA) being administered methotrexate. The results suggest that the combination of needle fear, impact of methotrexate treatment, and procedural consequences, e.g., blood sampling, all contribute to the distress and the loss of quality of life of children with JIA. Remarkably, no studies examining fear of injections or injection pain in children with rheumatic diseases receiving biologicals were identified.

*Conclusion*: Strategies to optimize administration of disease modifying anti-rheumatic drugs should be systematically investigated.

**Table:** 

**What is Known:** • *Repeated parenteral administration of drugs is burdensome for children with rheumatic diseases.*	
**What is New:** • *Needle fear should be investigated systematically to optimize administration of disease-modifying anti-rheumatic drugs.*

**Electronic supplementary material:**

The online version of this article (10.1007/s00431-018-3286-8) contains supplementary material, which is available to authorized users.

## Introduction

Intravenous (IV), intramuscular (IM), and subcutaneous (SC) injections are the three most frequently used procedures to administer parenteral medication, accounting for 16 billion injections worldwide on an annual basis [29]. Injections are often perceived as dolorous and pain during injections can lead to the development of needle phobia or disproportionate amounts of fear or vasovagal syncope when presented with needles.

Fear of needles or injections is a common problem in the general population [21, 23, 25, 30] and typically begins in childhood [2, 3, 22]. There is an inverse correlation between age and the extent of needle pain and fear [1, 11, 30]. Major reasons are the increasing tolerance for pain with aging and the frequency of needle procedures during childhood [8, 19]. Even the most extreme form of needle fear, blood-injury-phobia, is not uncommon; its estimated lifetime prevalence is 3.2–4.5% [3, 15], which likely is an underestimation as patients with needle phobia tend to avoid health care [9, 13, 17]. Needle fear is a documented barrier to immunization in children [25].

Children and adolescents with rheumatic diseases may be particularly vulnerable to the long-term consequences of procedure-related pain like injection pain, due to their often young age at diagnosis and long duration of parenteral treatment with anti-rheumatic drugs. This medication is given intravenously or subcutaneously, every month, week or, as for children with systemic juvenile idiopathic arthritis (JIA), even every day. Children receiving treatment with injectable drugs in the home setting frequently need to be kept in position by one of their parents or caregivers while the other parent is administering the injection. These frequent painful injections are an important stress factor for many children and their families and therefore a recurrent topic during outpatient consultations. Especially for children receiving daily anakinra medical care at home can be helpful, although this comes not without added financial costs.

We were interested if the perceived high burden of treatment and development of needle phobia and its effect on long-term treatment adherence in children with rheumatic diseases had been quantified. Therefore, a literature review was performed systematically, focusing on children receiving treatment with disease- modifying anti-rheumatic drugs (DMARDs).

## Methods

A systematic search for studies on fear of needles or injection pain in children was conducted together with a librarian. The following databases were accessed: PubMed, Embase, Web of Science, and the Cochrane Library (Appendix 1 Search queries). Duplicates were removed manually. Two authors reviewed the remaining abstracts for eligibility. Meeting abstracts were not considered for inclusion. Studies reporting on children with rheumatic diseases needing treatment with DMARDs were included. Studies regarding antibiotic prophylaxis in the context of rheumatic heart disease were excluded. As only a few abstracts were eligible, study selection was solely based on the content and no formal quality assessment was done.

## Results

The search resulted in 973 unique abstracts (February 20, 2018). Two relevant studies were identified, both about children with JIA taking methotrexate [20, 27]. No relevant studies regarding biologicals were identified. Methotrexate is the first-choice DMARD for JIA. Common side effects of methotrexate are gastrointestinal symptoms such as nausea and vomiting, which can partially be prevented by supplying folic acid. Children who receive methotrexate frequently develop anticipatory nausea and vomiting [5]. Methotrexate can be administered orally and subcutaneously.

In the study by Mulligan and colleagues, a questionnaire was completed by a mother proxy for 171 children with JIA (mean age 9.0 years, SD 4.0 years, 72% female) taking methotrexate for at least 6 months [20]. The questions on needle-related problems were on anxiety about injections and blood tests experienced by the children during the month before, with the answers never, almost never, sometimes, often, or almost always [20, 28]. Over a third of the children had fear of injections and/or blood tests often or almost always. Half of the children experienced these feelings at least sometimes. Anxiety about injections but not blood tests was more frequently observed for children with subcutaneous injections than for children receiving oral methotrexate. A younger age and a shorter treatment duration were related to anxiety about blood tests. Anxiety about blood tests was a significant independent predictor of poorer scores on a psychosocial score. Anxiety about injections was related to younger age, subcutaneous methotrexate administration, and higher disease activity. Anxiety about injections did not affect treatment adherence.

Van der Meer et al. interviewed the parents of ten children with JIA (mean age at start methotrexate 6.7 years, range 2.8–12.9 years, 90% female) receiving methotrexate who had been referred to a psychologist, and the parents of an additional 19 patients (mean age at start methotrexate 7.3 years, range 1.3–13.4 years, 76% female) with JIA also using methotrexate. Nine out of the ten children who were referred for behavioral therapy, and eight of the other group, showed panic and distress in the anticipation of methotrexate treatment. The behavioral distress was observed in the children treated with injections as well as in the children receiving oral methotrexate. The authors describe refusal of methotrexate administration by children, but do not provide quantitative data.

## Discussion

In the clinical setting, the focus of clinicians is often geared towards modifying and improving diseases by assessing risk and benefit. However, little is known about the impact on the quality of life of young children that are diagnosed with JIA and need to start with repeated, often long-lasting or life-long invasive therapies. While this is a very relevant topic, we identified a gap in literature which had not been appropriately addressed to date. Therefore, we systematically investigated the burden of parenteral repeated administration in children.

Pain is a common stressor in children with rheumatic diseases and is most commonly due to arthritis [12]. Clinical experience shows that young children with arthritis frequently do not show pain. Rather, their parents observe a swollen joint, e.g., knee or ankle, or limping. Older children with arthritis do more often express feeling pain. Even with stable JIA, pain remains a major problem and has a significant impact on quality of life [4, 7, 16, 18, 24]. It might seem logical to assume that children experiencing vast amounts of pain develop a higher pain threshold. However, patients with JIA have a lower threshold for experimentally induced pain than healthy controls [10, 16, 26]. This altered pain threshold is observed for both the affected joints as well as areas unaffected by the disease and persists after sensory neuronal input from the injured site. Perception of pain is multifactorial and it is unknown if the altered pain threshold in children with JIA has predominant physiological or psychological causes. However, it is recognized that both central and peripheral sensitization play a role in children with juvenile chronic arthritis [14]. The resulting altered pain threshold emphasizes even more the need to minimize procedural related pain.

The studies by Mulligan et al. and Van der Meer et al. show that distress associated with methotrexate administration, both orally and subcutaneous, negatively impacts the quality of life of children with JIA. The interpretation of these studies in the context of needle fear is complicated due to methotrexate associated anticipatory nausea and vomiting. The combination of needle fear, impact of methotrexate treatment, and procedural consequences, e.g., blood sampling, all contribute to the distress, and the loss of quality of life of children with JIA.

While our findings corroborate common knowledge, it is striking how little research and established evidence about scope and scale of needle fear is available. Interpretation of the literature is complicated due to the inconsistent use of terms relating to the stressors associated with therapeutic drug injections. Many questions remain to be answered regarding the perceived burden of painful injections and needle fear in children with rheumatic diseases requiring treatment with DMARDs. First, the effect of procedural pain, i.e., injection pain, might be better studied in children receiving treatment with biologicals rather than children receiving methotrexate as the results might be confounded by anticipatory vomiting associated with methotrexate. No literature was identified which addressed this issue. Moreover, chronic therapy might affect the perceived burden over time; therefore, it could be worthwhile to longitudinally study the anxiety about injections from the start of treatment with biologicals. Also, it may be worthwhile to develop standardized, age-related tools to uniformly quantify the untoward injection-related phenomena. Future studies should use well-defined outcome parameters to systematically evaluate the different factors contributing to the decrease in quality of life due to therapeutic drug injections. Last, it might be insightful to compare procedural pain in children receiving treatment with DMARDs compared to healthy, age-matched controls, for example in the context of vaccination. Ideally, the aspects of comfort during the administration of drugs in pediatrics should be evaluated during the drug development plan. These aspects of comfort include the route of administration, frequency, and tolerance [6].

After performing an impact analysis, new strategies might be developed to optimize administration of DMARDs to children. This aspect of health care might represent a financially less attractive target than the development of new drugs. However, ultimately, there is ample room to improve the health-related quality of life of pediatric patients with rheumatic diseases by minimalizing chronic stressors, like procedure-related pain.

## Electronic supplementary material

ESM 1 (DOCX 13.1 kb)

## Abbreviations

DMARDs: Disease-modifying anti-rheumatic drugs
IM: Intramuscular
IV: Intravenous
JIA: Juvenile idiopathic arthritis
SC: Subcutaneous
