# Supplementary material for: The effect of repeated methotrexate injections on the quality of life of children with rheumatic diseases
Source: Eur J Pediatr. 2018 Nov 17;178(1):17–20. doi: 10.1007/s00431-018-3286-8 (PMC6311192; doi:10.1007/s00431-018-3286-8)
Supplement: Supplementary file 1 — (DOCX 13.1 kb) [file 431_2018_3286_MOESM1_ESM.docx]

**Appendix 1 Search queries**

PubMed

**(("needle phobia"[tw] OR "needle phobias"[tw] OR "needle phobic"[tw] OR "needle phobics"[tw] OR "fear of needle"[tw] OR "fear of needles"[tw] OR "needle anxiety"[tw] OR "needle pain"[tw] OR "needle fear"[tw] OR "needle fearful"[tw] OR "needle fearing"[tw] OR "needle fears"[tw] OR "fear of injection"[tw] OR "fear of injections"[tw] OR "injection fear"[tw] OR "injection fearful"[tw] OR "injection fears"[tw] OR "injection anxiety"[tw] OR "injection pain"[tw] OR "injection pains"[tw] OR (("Anxiety"[majr] OR "Fear"[majr] OR "Phobic Disorders"[majr]) AND ("Needles"[majr] OR "Injections"[majr])) OR (("Anxiety"[ti] OR "Fear"[ti] OR Phobi*[ti]) AND ("Needles"[ti] OR "Injections"[ti] OR "Needle"[ti] OR "Injection"[ti]))) AND ("Child"[mesh] OR "Infant"[mesh] OR "Adolescent"[mesh] OR "Child"[tw] OR "Infant"[tw] OR "Adolescent"[tw] OR "Children"[tw] OR "Infants"[tw] OR "Adolescents"[tw] OR "Adolescence"[tw] OR "juvenile"[tw] OR pediatr*[tw] OR paediatr*[tw] OR schoolchild*[tw] OR "girl"[tw] OR "girls"[tw] OR "boy"[tw] OR "boys"[tw]) AND english[la])**

Embase

**(("needle phobia".mp OR "needle phobias".mp OR "needle phobic".mp OR "needle phobics".mp OR "fear of needle".mp OR "fear of needles".mp OR "needle anxiety".mp OR "needle pain".mp OR "needle fear".mp OR "needle fearful".mp OR "needle fearing".mp OR "needle fears".mp OR "fear of injection".mp OR "fear of injections".mp OR "injection fear".mp OR "injection fearful".mp OR "injection fears".mp OR "injection anxiety".mp OR *"injection pain"/ OR "injection pain".ti OR "injection pains".ti OR ((exp *"Anxiety"/ OR exp *"Fear"/ OR exp *"Phobia"/) AND (exp *"Needle"/ OR exp *"Injection"/)) OR (("Anxiety".ti OR "Fear".ti OR Phobi*.ti) AND ("Needles".ti OR "Injections".ti OR "Needle".ti OR "Injection".ti))) AND (exp "Child"/ OR exp "Infant"/ OR exp "Adolescent"/ OR "Child".mp OR "Infant".mp OR "Adolescent".mp OR "Children".mp OR "Infants".mp OR "Adolescents".mp OR "Adolescence".mp OR "juvenile".mp OR pediatr*.mp OR paediatr*.mp OR schoolchild*.mp OR "girl".mp OR "girls".mp OR "boy".mp OR "boys".mp) AND english.la)**

**Web of Science**

**((ts=("needle phobia" OR "needle phobias" OR "needle phobic" OR "needle phobics" OR "fear of needle" OR "fear of needles" OR "needle anxiety" OR "needle pain" OR "needle fear" OR "needle fearful" OR "needle fearing" OR "needle fears" OR "fear of injection" OR "fear of injections" OR "injection fear" OR "injection fearful" OR "injection fears" OR "injection anxiety" OR "injection pain" OR "injection pains") OR ti=(("Anxiety" OR "Fear" OR "Phobia") AND ("Needle" OR "Injection")) OR ti=(("Anxiety" OR "Fear" OR Phobi*) AND ("Needles" OR "Injections" OR "Needle" OR "Injection"))) AND ts=("Child" OR "Infant" OR "Adolescent" OR "Child" OR "Infant" OR "Adolescent" OR "Children" OR "Infants" OR "Adolescents" OR "Adolescence" OR "juvenile" OR pediatr* OR paediatr* OR schoolchild* OR "girl" OR "girls" OR "boy" OR "boys") AND la=english) NOT ti=(veterinary OR rabbit OR rabbits OR animal OR animals OR mouse OR mice OR rodent OR rodents OR rat OR rats OR pig OR pigs OR porcine OR horse* OR equine OR cow OR cows OR bovine OR goat OR goats OR sheep OR ovine OR canine OR dog OR dogs OR feline OR cat OR cats))**

**Cochrane**

**((("needle phobia" OR "needle phobias" OR "needle phobic" OR "needle phobics" OR "fear of needle" OR "fear of needles" OR "needle anxiety" OR "needle pain" OR "needle fear" OR "needle fearful" OR "needle fearing" OR "needle fears" OR "fear of injection" OR "fear of injections" OR "injection fear" OR "injection fearful" OR "injection fears" OR "injection anxiety" OR "injection pain" OR "injection pains"):ti,ab,kw OR (("Anxiety" OR "Fear" OR "Phobia") AND ("Needle" OR "Injection")):ti OR (("Anxiety" OR "Fear" OR Phobi*) AND ("Needles" OR "Injections" OR "Needle" OR "Injection")):ti) AND ("Child" OR "Infant" OR "Adolescent" OR "Child" OR "Infant" OR "Adolescent" OR "Children" OR "Infants" OR "Adolescents" OR "Adolescence" OR "juvenile" OR pediatr* OR paediatr* OR schoolchild* OR "girl" OR "girls" OR "boy" OR "boys"):ti,ab,kw)**
